# “The Two Brothers”: Reconciling Perceptual-Cognitive and Statistical Models of Musical Evolution

**DOI:** 10.3389/fpsyg.2018.00344

**Published:** 2018-04-04

**Authors:** Steven Jan

**Affiliations:** Music and Drama, University of Huddersfield, Huddersfield, United Kingdom

**Keywords:** qualitative, quantitative, perceptual-cognitive, statistical, memetics, phylomemetics, cultural evolution

## Abstract

While the “units, events and dynamics” of memetic evolution have been abstractly theorized (Lynch, 1998), they have not been applied systematically to real corpora in music. Some researchers, convinced of the validity of cultural evolution in more than the metaphorical sense adopted by much musicology, but perhaps skeptical of some or all of the claims of memetics, have attempted statistically based corpus-analysis techniques of music drawn from molecular biology, and these have offered strong evidence in favor of system-level change over time (Savage, 2017). This article argues that such statistical approaches, while illuminating, ignore the psychological realities of music-information grouping, the transmission of such groups with varying degrees of fidelity, their selection according to relative perceptual-cognitive salience, and the power of this Darwinian process to drive the systemic changes (such as the development over time of systems of tonal organization in music) that statistical methodologies measure. It asserts that a synthesis between such statistical approaches to the study of music-cultural change and the theory of memetics as applied to music (Jan, 2007), in particular the latter's perceptual-cognitive elements, would harness the strengths of each approach and deepen understanding of cultural evolution in music.

## 1. Introduction: approaches to the study of cultural evolution

The dichotomy, even tension, between qualitative and quantitative research methods aligns to some extent with the “two cultures”—the artistic/humanistic and the scientific, respectively—famously outlined by Snow (1964)^1^. While this is certainly an oversimplification—the two approaches often blend; and both may be deployed in the service of falsifiability (Popper, 1959), the acid test of a scientific theory—it has, until quite recently, largely been the norm, certainly in western musicology^2^. Nevertheless, the explosive growth in computer power, and its increasing accessibility, has, over the last two decades, put systematic approaches in the hands of scholars in the arts and humanities. In music research, such approaches are typified by the interest in “empirical [experimental, data-rich] musicology” (Cook, 2004) and, more broadly, by the current attention paid in the humanities to the promises of “big data” (Sharma et al., 2014), which allows, for instance, for large-scale statistical analysis of music-related bibliographical data (Rose et al., 2015).

Conversely, a number of research traditions in the sciences have used music data in quantitative studies, including the Music Information Retrieval Exchange (MIREX) project^3^. This work stems partly from an interest in how technology can expedite music research—particularly in the fields of pattern-finding and data-retrieval—and partly from a recognition that the inherent complexity of music makes it a singular challenge for the design and implementation of computerized analytical tools. A similar motivation underpins cognitive science in music: often, its music-orientated practitioners pursue it in order to try to unravel the mysteries of the art form; whereas its science-orientated researchers wish to understand the deep embeddedness of music in multiple brain and body systems (Schulkin, 2013). Linking data-searching and analysis and cognitive science, the recent development of systems which autonomously create music—what might be termed the computer simulation of musical creativity—is testament to the power of computers to bring together research in music, artificial intelligence and cognitive science in the service of understanding what still seem to be the mysteries of creativity (Miranda et al., 2003; Boden, 2004), whether this research is motivated by artistic/humanistic or by scientific impulses^4^.

The study of cultural evolution has been approached from both of Snow's perspectives. From the scientific, there is a tradition of research at the interface of anthropology, sociology and evolutionary biology which uses broadly Darwinian methods to understand the spread of cultural items, including ideas, artistic traditions and artifact-manufacturing technologies (Cavalli-Sforza and Feldman, 1981), this cultural transmission sometimes being correlated with genetic transmission (Shennan, 2002). From the artistic/humanistic, there is a long tradition of research (conducted broadly under the rubric of historical musicology) of referring to change in music as in some sense evolutionary (Perry, 2000). But this ascription is largely metaphorical; that is, it documents morpho-stylistic changes—in the outputs of composers, in the development of genres, or in the cultures of places or times—but it does not argue for a Darwinian (or any other algorithmic) basis as the mechanism driving this change. As an artistic/humanistic field, it clearly does not want to deny the agency of the composer, however that is understood to arise (Blackmore, 2010), just as composers do not want to deny it of and for themselves^5^.

By contrast, many would argue that because musical patterns, however defined, manifestly demonstrate variation, inheritance (transmission) and selection—“principles [which] apply equally to biological and cultural evolution” (Savage, 2017, p. 9)—they conform to Darwin's theory of evolution by (natural) selection^6^. That is, such patterns—Dawkins' *memes*—instantiate the evolutionary algorithm, but are sequences of elements in cultural media—such “phemotypic” (extra-somatic) products as “tunes, ideas, catch-phrases, clothes fashions, ways of making pots or of building arches” (Dawkins, 1989, p. 192), which devolve to “memotypic” patterns of neuronal interconnection (Calvin, 1996; Jan, 2011; Mhatre et al., 2012)—rather than biological-medium (DNA) sequences^7^. In this sense, “music literally evolves …[because] musical evolution follows patterns and processes that are similar, but not identical, to [those of] genetic evolution” (Savage, 2017, pp. 38, 22).

Accepting the memetic formalization of cultural evolution as real and not metaphorical, and using a small case study which, it is hoped, can be scaled and generalized, this article attempts to reconcile approaches drawn from the perceptual-cognitive and the statistical domains as they apply to the evolution of music^8^. It regards these two domains as broadly aligning, respectively, with the qualitative/quantitative distinction discussed above, although it recognizes that the perceptual-cognitive is of course formalizable and measurable (and thus partly quantitative/statistical) using the methodologies of cognitive science. In this sense, the article emphasizes the perceptual-cognitive/statistical dichotomy as arguably more meaningful for the understanding and advancement of memetics than the qualitative/quantitative.

Section 2 discusses some of the criticisms of memetics, arguing in its defense that its central claims, grounded as they are in important psychological principles, cannot be lightly dismissed. Section 3 discusses how relationships between musical patterns can be formalized using a combination of perceptual-cognitive and statistical approaches in ways that offer a robust model for the development of memetics. Section 4 follows up some implications of memetic similarity measurements, considering the representation of evolutionary relationships using taxonomic trees. Section 5 looks forward to the future integration of perceptual-cognitive and statistical approaches using computer technology.

The article offers two principal claims. The first of these is as follows: a purely statistical approach based on counting note-edits without consideration of perceptual-cognitive aspects gives an incomplete account of cultural evolution. A second, derived, claim will be outlined at the start of section 3.

## 2. The problem with memetics?

To demonstrate the nature-culture similarities he hypothesizes, Savage (2017) uses techniques drawn from molecular genetics—discussed more fully in section 3—to compare the basic mutational-editing operations of note conservation, substitution, insertion and deletion (Savage, 2017, p. 53) in corpora of folk-song melodies with protein modification in biological transmission. He argues that an advantage of a “rigorously quantitative approach modeled on molecular genetics is that such quantitative approaches have shown success in rehabilitating cultural-evolutionary theory after much criticism of earlier incarnations such as Dawkins' “memetics”' (Savage, 2017, p. 45).

Criticism of memetics—Gould called it a “meaningless metaphor” (in Blackmore, 1999, p. 17; see also Kuper, 2000)—has arguably been counterbalanced by as much endorsement (Dennett, 2007), or at least by the acceptance that some problems in cultural studies are readily addressed by recourse to memetics. Yet Savage is to some extent correct in his implication that a fault with memetics (assuming one accepts its fundamental premises) is that it has hitherto been formulated in a somewhat imbalanced way, with too much emphasis on the qualitative and too little on the quantitative (but see McNamara, 2011). In the terms of section 1, it might therefore be believed that it has not (yet) been formulated in such a way as to be falsifiable. Yet this is to ignore the work of several scholars who have attempted to use the insights of memetics in quantitative studies (Adamic et al., 2014); and also, perhaps more importantly, to discount the work of Lynch (1998), who has arguably made the greatest contribution to the formalization of memetics, even though his models, to my knowledge, have not yet been systematically applied or tested^9^.

If Savage's (2017) criticism of memetics as insufficiently orientated toward quantitative methodologies is accepted, then it is surely valuable that the more qualitative insights of memetics—often based upon introspective evaluation of the nature of certain musical patterns and their transmission across cultural time and space—are supported by quantitative work which counts and measures such phenomena systematically. This, by its very nature, implies statistical studies of large corpora. Nevertheless, the danger with such approaches, particularly the type of molecular-genetics approach adopted by Savage and his collaborators, is that they risk being focused on too low a descriptive level and may arrive at statistical generalities rather than meaningful particularities—the former an approach not dissimilar to the “beanbag genetics” criticized by Mayr (Dronamraju, 2010; but see also Juhász and Sipos, 2010). Savage and Atkinson (2015) concede this, arguing for the importance of taking into account:

higher-level units of musical structure and meaning. In music, as in genetics, the individual notes that make up the sequences have little meaning in themselves. The phylogenetic analysis of sequences is thus merely the starting point from which to understand how and why these sequences combine to form higher-level functional units (e.g., motives, phrases) that co-evolve with their song texts and cultural contexts of music-making as they are passed down from singer to singer through centuries of oral tradition (Savage and Atkinson, 2015, p. 167).

In this sense, it is important to consider—in the terms of the long-running debate in biology—the relevant *units of selection* (Lewontin, 1970), which requires a degree of nature-culture mapping^10^. While the protein sequences which Savage (2017) takes as analogous to musical sequences are useful exemplars of mutational operations, they have little evolutionary meaning in themselves. This is because *genes* are selected for, not nucleotides, nor, in Savage's case, the amino acids which make up the proteins whose production genes code for. Concomitantly, by focusing on discrete pitches—equated by Savage with the component amino acids of proteins—one is neglecting psychologically meaningful *groups* of pitches—these, in Savage's terms, equating to genes, which Dawkins regards as “any portion of chromosomal material that potentially lasts for enough generations to serve as a unit of natural selection” (Dawkins, 1989, p. 28). The mappings posited by Savage (2017) are summarized in Table 1, the first and second columns representing Savage's molecular-genetic mapping of (bio)chemical and musical structure, and the third and fourth columns representing a mirror-image, memetically motivated set of mappings (see also Jan, 2013, p. 152, Figure 1).

**Table 1:** Nature-culture mappings.

**Molecular-genetic**		**Memetic**	
**(Bio)chemistry**	**Music**	**Music**	**(Bio)chemistry**
Amino acid	Single pitch	Museme-element	Atom
?	Motive	Museme	Molecule
Protein	Musical phrase	Museme sequence/Musemeplex (see section 3)	Multi-molecule complex

**Figure 1:**
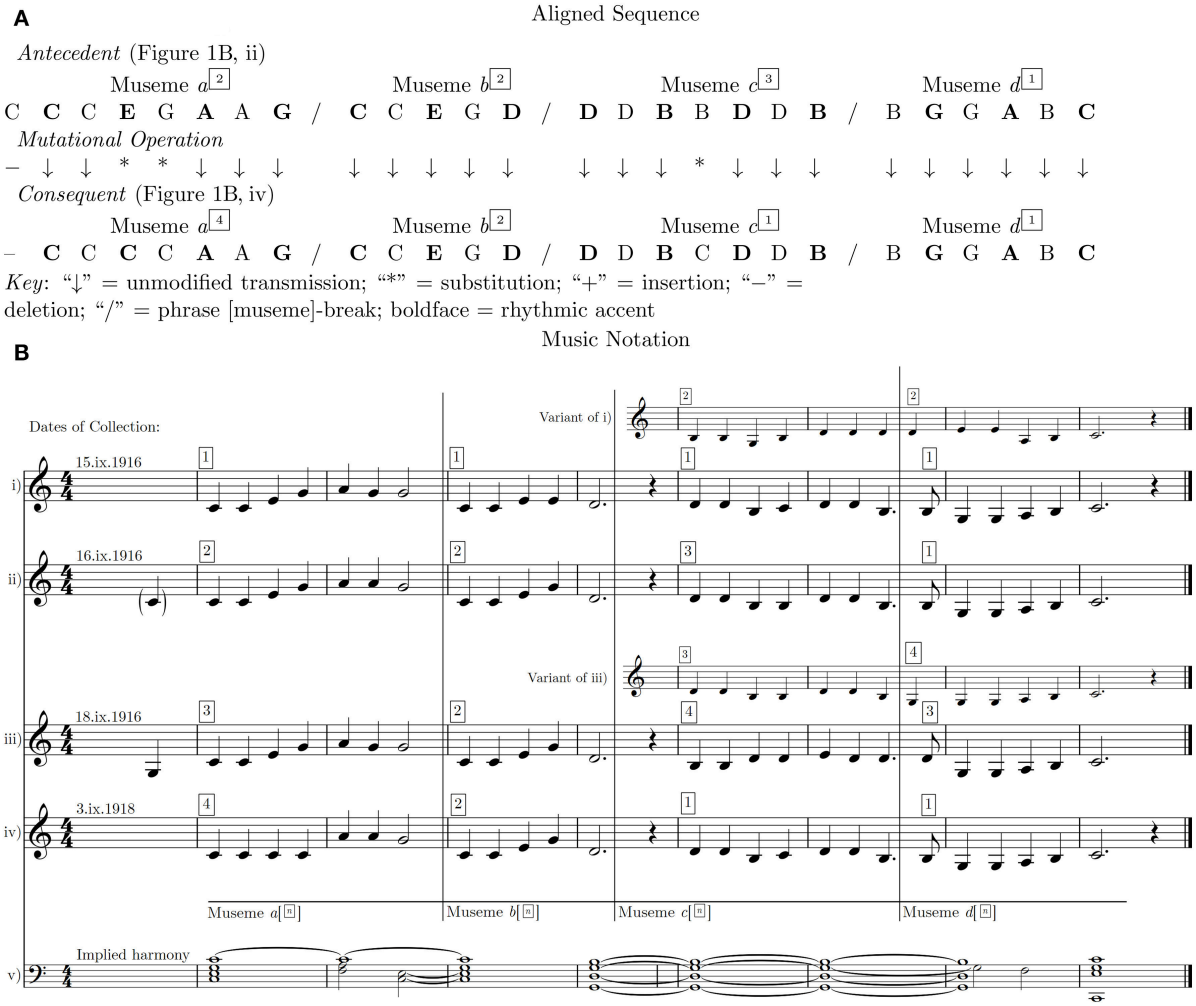
Mutation in aligned pitch-sequences: “The Two Brothers”.

Thus, Savage's (2017) positing that amino acids are equivalent (in some abstract sense) to individual pitches and that proteins are equivalent to melodies is problematic because melodies are often made up of a number of discrete intermediate-level patterns—*musemes* (music-memes), in my terminology, and motives in Savage's (2017)—a crucial cognitive level which is not explicitly accounted for (hence the “?” in Table 1) in his approach. By “museme”—a particularly salient example of which is the opening four notes of Beethoven's Fifth Symphony—is meant a perceptually-cognitively-demarcated melodic/horizontal (pitch-rhythm) and/or harmonic/vertical collection which is capable of being retained in short-term memory and which possesses “just sufficient copying-fidelity to serve as a viable unit of [cultural] selection” (Dawkins, 1989, p. 195)^11^.

Such groups of pitches—the gene-equivalent patterns theorized by memetics—are much stronger candidates for the units of selection in cultural evolution than Savage's isolated pitches. This is because a m(us)eme is not a m(us)eme unless, as Dawkins states, it *can* act as a unit of selection. To serve this function it has to have a *discrete identity*; that is, it must (i) be discrete (demarcated to some extent from the patterns surrounding it, even if it partially overlaps with them; Jan, 2007, p. 74); and it must (ii) have an identity (it must have some attribute(s) which distinguish it to some extent from other, similarly demarcated, patterns and which motivate(s) its copying). These two points allow us to understand memetic selection as success in the competition for the finite attention and memory resources of a m(us)eme's potential human hosts.

There is very strong evidence from the cognitive-psychological literature that music is perceived in terms of such melodic/harmonic groups; and it would appear that they derive, in part, from the phenomenon of expectation (anticipation, prediction) (Huron, 2006; Husserl, 2013). As with many music-related perceptual-cognitive processes, this is a consequence of both bottom-up (innate/genetically determined) and top-down (learned/memetically determined) factors (Narmour, 1990). While subject to innate constraints, often considered under the rubric of Gestalt psychology, much of our perception of music (and indeed language) relies upon the statistical learning of conventions as a result of enculturation (Gjerdingen, 1988; Byros, 2009). This process has been modeled in a number of computer simulations: discussing their Information Dynamics of Music (*IDyOM*) model, Pearce and Wiggins (2012) argue that violation of expectations leads not only to affective responses (Meyer, 1956), but is a significant force in imposing grouping boundaries. Moreover, both bottom-up and top-down factors regulate the selective environment of musemes, for the former dictate the constraints a museme must satisfy in order to be perceived, cognized and memorized (Lerdahl, 1992; Velardo and Vallati, 2016); while the latter include the totality of musemes within a cultural community (the *museme-pool*), against which a given museme must compete (in the sense outlined at the end of the previous paragraph).

To expand upon the foregoing, one can make the following points:

- *Bottom-up:* Evolutionarily selected predispositions to vocal learning (Merker, 2012) make humans very good at attending to musilinguistic sounds (Brown, 2000; Mithen, 2006; Fitch, 2010) and abstracting statistical regularities from them (Kirby, 2013). This abstraction is fostered by the imposition of grouping boundaries, which “are perceived before events for which the unexpectedness of the outcome (*h*) and the uncertainty of the prediction (*H*) are high” (Pearce and Wiggins, 2012, p. 638). Such grouping boundaries create the “chunking” (Snyder, 2000, pp. 53–56) necessary for processing by short-term memory.
- *Top-down:* Suitably packaged, this musical information is retained in individual and collective memories; indeed, it would not be retained if it were not delineated. It might be termed, after Chomsky, “I-music” (internal, brain-stored, music) and “E-music” (external, culture-stored, music), respectively (Fitch, 2010, p. 32). Chunked musical patterns also influence the perception of other patterns, including their grouping, because “that which is copied [retained in memory] may serve to define the pattern” (Calvin, 1996, p. 21; see also Jan, 2011, section 4.1).

More broadly, the bottom-up/top-down duality raises the issue of gene-meme coevolution (Durham, 1991), because it pits biological replicators against their cultural equivalents. At the highest level, *system-orientated* research in coevolution encompasses the evolution of the human capacity for musicality and other phenotypic attributes (Blackmore, 2000, pp. 31–34; Jablonka and Lamb, 2014; Podlipniak, 2017); but *replicator-orientated* research in this area is generally not conducted with a specifically memetic orientation (but see Shennan, 2002), tending to focus on gene-level changes driven by (often generic) cultural pressures (Richerson et al., 2010). Thus, future research in coevolution might attempt to investigate meme-level changes driven by (specific) genetic pressures, and the interactions between specific memes and genes.

Given the foregoing, while the statistical data on folk-song corpora edits of Savage (2017) are strong evidence in favor of cultural evolution, they should be regarded as *epiphenomena* of musemic-evolutionary processes—consequences of the changes which occur when discrete musical patterns are transmitted with copying errors and are differentially selected. To gain a deeper understanding of such statistical data, one must regard the mutational changes (conservation, substitution, insertion and deletion) as forces not only *driving* musemic mutation and, ultimately, musico-stylistic evolution (Jan, 2015), but also as forces *constrained by* the psychological realities of pattern-formation and propagation. That is, one must take into account two countervailing forces: (i) susceptibility to mutational pressures (perhaps engendered by weak perceptual-cognitive demarcation and/or low intra-museme coherence) may distort a museme (resulting in high entropy; Margulis and Beatty, 2008), but may introduce a variant which has a higher perceptual-cognitive salience (Berlyne, 1971; Martindale, 1986), and therefore potentially greater replicative prospects, than its antecedent—Dawkins' “fecundity” (Dawkins, 1989, p. 194); and (ii) resistance to mutational pressures (perhaps engendered by strong perceptual-cognitive demarcation and/or high intra-museme coherence) may preserve multiple copies of a museme (resulting in low entropy), and may therefore foster an increase in its representation in the museme-pool over time—Dawkins' “copying-fidelity” (Dawkins, 1989, p. 195).

Lastly, one might argue that a memetic orientation erodes the qualitative-quantitative distinction—or, rather, that it allows us to understand it as a continuum—because it supports a range of methodologies from (qualitative) assessments of the aesthetic effects of certain musemes in particular musical contexts to (quantitative) measurements of museme frequency and transmission relationships.

## 3. Quantification of evolutionary distance in musemes

To the first claim outlined in section 1—that a purely statistical approach based on counting note-edits without consideration of perceptual-cognitive aspects gives an incomplete account of cultural evolution—a second has arisen from section 2: that statistical data derived from measuring mutational changes, while illuminating, are epiphenomena of musemic evolution.

To investigate this, I consider some of Savage's (2017) specific data in a small case study, attempting to relate them to the musical patterns from which they arise. It is important to note at this stage that the tracking of conservations, substitutions, insertions and deletions is done partly in the service (in one of his studies) of grouping folk songs into tune-families (Cowdery, 1984), and I will focus on examples from one sub-family which will hopefully serve as a microcosm of more general issues. This focus is perhaps characteristic of the qualitative (“less is more”)/quantitative (“more is more”) distinction.

Figure 1 shows one such melody, “The Two Brothers”, no. 49 of the “Child Ballads”, two variants of which are incorporated by Savage in his dataset. The Child Ballads are a collection of British folk ballads (specifically, their lyrics), assembled (some from American sources) by Child (1904). The (often diverse) melodies associated with these lyrics were later collated and categorized by Bronson (1959). This particular ballad, originally from Scotland, concerns the death—variously accidental or intentional—of one of the eponymous school-age brothers by the other's knife, and the deceased boy's subsequent interment^12^.

What I label the “Antecedent” in Figure 1A (Figure 1Bii) was transcribed in Bronson's (1959) sources from a rendition by “Mrs. Ellie Johnson (23), Hot Springs, N.C., September 16, 1916” (Bronson, 1959, p. 391, no. 16); and the “Consequent” in Figure 1A (Figure 1Biv) from a rendition by “Mrs. Lucindie (G.K.) Freeman, Marion, N.C., September 3, 1918” (Bronson, 1959, p. 390, no. 15). Phrase-ending marks (represented by continuous vertical lines in Figure 1B) are Bronson's and are retained by Savage (2017). Being clear points of articulation, these marks are equivalent to the terminal nodes of the four musemes—Musemes (hereafter “M”) *a*–*d*—which constitute these melodies (labeled under Figure 1Biv)^13^. While Savage is correct in labeling these two versions as “older” and “younger” (in terms of date of collection), respectively, there are actually four melodies in this group (six if one includes the variants in the second halves of two of them), and his “older” is not the “oldest”: this status goes, by one day, to Figure 1Bi^14^. Figure 1Bv, represents the implied harmony of these melodies, which may or may not have been realized in some performances, perhaps on guitar.

While it makes sense methodologically for Savage (2017) to think in terms of “older” (antecedent) and “younger” (consequent) patterns, the fact that: (i) the time intervals between the recording of these phemotypic forms are so short (three days, in the case of Figures 1Bi–iii); (ii) the individuals concerned would presumably have assimilated these melodies months or years before the date of collection; and (iii) the geographical area from which they were collected is relatively constrained (the western counties of North Carolina, with two of the four melodies being collected in the same town, Hot Springs), all suggest that a model of linear transmission in collection-date order, with clearly demarcated, sequential mutations, is obviously highly improbable. This conclusion is further reinforced by the fact that the variants in Figures 1Bi,iii, were presumably recorded on the same occasions as the ostensibly “principal” form. Given these points, references in what follows to “earlier”/“antecedent” and “later”/“consequent” forms of melodies and musemes must be understood as relating only to the dates of collection and to the resultant numeration in Figure 1B, and not as hypotheses of evolutionary descent-order.

An arguably more realistic model would be of an ecosystem in which a relatively stable framework—defined by balanced and rhyming periodicity, implied harmony, cadence patterns and axial pitches—was generated by means of a number of interchangeable musemes being repeatedly co-replicated. This framework is eight bars in duration, with a I–V; V–I two-phrase/four-sub-phrase structure and a “middle cadence …on the supertonic [$\hat{2}$, supported by an implied V]” (Bronson, 1959, p. 384). It is clearly not unique to this set of song variants: it forms the basis, much expanded, of “two-phrase”/“balanced” binary form (Rosen, 1988, p. 22; Hepokoski and Darcy, 2006, p. 355), as well as of numerous other folk-song melodies (Bronson, 1959, p. xii)^15^. It serves as a container for a set of musemes which were interchangeable in ways which did not compromise the integrity of the melody, as understood by members of the cultural community which replicated it in conjunction with a similarly variable set of verbal-conceptual (lyric/text) memes.

In this sense, “The Two Brothers” is a higher-order structure re-instantiated/generated by the repeated re-conglomeration of a set of functionally equivalent musemes, each of which serves to articulate a specific node of the structure. The notion of functionally analogous musemes is essentially that of the replicator *allele* (Dawkins, 1983a, p. 283). This concept, when used in the context of cultural evolution, refers to musemes which are similar in their basic structure and/or function, such that members of the same museme *allele-class* are interchangeable—in the sense of being equally viable and coherent—in a specific context (such as a certain point in a phrase or a particular modulatory juncture, etc.) (Jan, 2016). The framework/higher-order structure referred to above might be termed a *musemeplex*—i.e., a complex formed by the repeated co-replication of a set of musemes (in the case of Figure 1B, M*a*–M*d*) which are nevertheless also individually replicated (Jan, 2007, p. 80). Automatically, the replication of a musemeplex results in the replication of what might be termed a *musemesatz*—i.e., a shallow-middleground-level structure, the “skeleton” of a musemeplex, generated by the tendency of a set of allelically related musemes to conglomerate in broadly similar ways in two or more contexts (Jan, 2010). As represented in Figure 1, allele-identifiers are shown as superscript boxed Arabic numbers (assigned according to date of collection), so that (for example) bb. 1–2 of Figure 1Bi, is labeled Allele $\begin{array}{c} 1 \end{array}$ of M*a*, symbolized hereafter in the text as “M*a*^$\begin{array}{c} 1 \end{array}$^”^16^.

Given this nexus of similarity relationships linking six melodies assembled from a set of fourteen alleles, how might we understand the connections between the component musemes and attempt to reconstruct their transmission relationships? Perhaps it is necessary to concede that one cannot ultimately reconstruct the nexus of transmission that gave rise to these six melodic variants, simply because human culture is so interconnected—and was even when these songs were current, in the pre-internet age—and the cultural interactions with which we are concerned were largely undocumented. But one might still try to sketch out possible evolutionary trajectories and develop methodologies which might be applicable to these and other cases. One way is to attempt to quantify the differences between them, in terms of measuring the mutational changes that separate them. Savage proposes the percent identity (PID) as a measure of evolutionary distance, this being defined as “the number of aligned positions (i.e., amino acids, DNA nucleotides, musical notes, etc.) that are identical (ID) divided by the sequence length (L).…We have chosen to use the average length of both sequences [L_1_, L_2_], as this appears to be the most consistent measure of percent identity” (Savage, 2017, pp. 53–54). This metric is represented in the following equation:^17^

(1) $$\begin{array}{r} PID=100\times \left\{\frac{\frac{ID}{L_{1}+L_{2}}}{2}\right\} \end{array}$$

Savage (2017) uses the PID as an index of the *mutational distance* between two variant *melodies* in order to assess a tune's membership of a particular tune-family—the larger the PID, the greater the likelihood of the melodies' belonging in the same tune-family. But there is no reason why this metric cannot also be used at the level of the *museme*, in order to quantify mutational distance between such patterns. Used this way, the PID may be used to assess membership of a museme allele-class (or, indeed, to investigate a relationship of presumed mutation which moves a museme from one allele-class into another). Membership of a museme allele-class implies—provided the musemes are of a comparable length—that the musemes in question are related by homology [“a character shared between two or more species that was present in their common ancestor” (Ridley, 2004, pp. 427, 480); what Darwin termed “descent with modification” (Darwin, 2008, p. 129)], rather than homoplasy [“a character shared between two or more species that was not present in their common ancestor” (Ridley, 2004, pp. 427–428, 480)]; that is, a relationship resulting from cultural transmission, rather than from “convergent evolution” (Ridley, 2004, p. 429), respectively. Nevertheless, as with comparable cases in biology, it is not always possible to decide with certainty which category specific cases belong in. While determination of a suitable PID threshold for perceptually-cognitively significant similarity might be achieved by means of empirical studies—whereby test musemes with various degrees of mutation are ranked by listeners according to their perceived relatedness—this would not necessarily permit the assignment of threshold-exceeding patterns to the same allele-class without fuller knowledge of the context of transmission.

A related metric is *mutation rate*, which is the number of “observed mutations per year” (Savage, 2017, p. 56), where the number of mutated pitches (*x*) is compared with the total number of pitches (*y*) over time (*t*). This is represented in the following equation:

(2) $$\begin{array}{r} MR_{\left(t\right)}=\left(x/y\right)/t \end{array}$$

Again, there is no reason why this metric cannot also be used at the museme level, in order to quantify the mutation rate between two museme alleles. While cultural evolution occurs at an absolute rate many orders of magnitude faster than biological evolution (Dawkins, 1989, p. 192), and indeed occurs at highly variable absolute rates (Savage, 2017, p. 107), if cultural evolution is scaled to biological evolution (i.e., if some relative rather than absolute mutation rate is considered), then the two processes may be broadly comparable. Mutation rate is directly correlated with “transmission fidelity” (Savage, 2017, p. 111), in that the lowest mutation rates are found in repertoires with high copying-fidelity, and *vice versa* (Dawkins, 1989, pp. 18, 194); these repertories tend, unsurprisingly, to be notationally (as opposed to orally) transmitted musics. In the case of these particular melodies, however, the time interval is so constrained, and the transmission nexus sufficiently unclear, for the mutation-rate metric to be of limited use (despite the illustrative calculation below) in the present context.

On this basis, the PID and MR values (the latter over a notional 2-year period, the time interval separating the collection of Figures 1Bii,iv) for M*a*^$\begin{array}{c} 2 \end{array}$^ and M*a*^$\begin{array}{c} 4 \end{array}$^ in Figure 1 are as follows:

(3) $$\begin{array}{r} PID=100\times \left\{\frac{\frac{5}{8+7}}{2}\right\}=71.4 \end{array}$$

(4) $$\begin{array}{r} MR_{\left(t\right)}=\left(3/8\right)/2=0.188 \end{array}$$

Because the musemes under investigation are components of a larger melody—they are, as argued above, independently replicated elements of a musemeplex which is transmitted, iso-sequentially ordered, as a collective—when the melody is copied from source to source, it is clear that the order and identity of musemes is either retained or obviously altered^18^. Such cases of musemic transmission are therefore more tractable—M*a*^$\begin{array}{c} 2 \end{array}$^ in one melody is clearly analogous to M*a*^$\begin{array}{c} 4 \end{array}$^ in a variant of that melody—than situations in which an isolated museme is potentially copied from an antecedent context (a piano sonata, for example) to a non-analogous consequent context (a symphony, for example). In the latter case, however, the PID and MR metrics might usefully be employed in order to assess the likelihood that a given pattern is indeed being transmitted from one context to another.

Such sequential-mapping constraints allow one to circumvent the fact that, at 71.4%, the PID value of M*a*^$\begin{array}{c} 2 \end{array}$^–M*a*^$\begin{array}{c} 4 \end{array}$^ in Figures 1Bii,iv is lower than the 85% Savage takes as an index of two melodies being “highly related” (Savage, 2017, p. 54)^19^. It is conceivable, however, that two melodies with a PID of this order of magnitude may not actually bear any obvious musemic relationships, owing to the insensitivity of the PID metric to museme similarity when the PID is calculated at the musemeplex (phrase) level (one might address this by calculating the PID at the musemeplex level using musemes rather than individual pitches as the units of measurement)^20^. Because Savage's (2017) ≥85% criterion applies to melodies, not musemes, and because his algorithm has paired the 71.4%-related M*a*^$\begin{array}{c} 2 \end{array}$^ and M*a*^$\begin{array}{c} 4 \end{array}$^ in Figures 1Aii,iv, there must by definition be a >85% similarity between the other musemes of the phrase, M*b*^$\begin{array}{c} n \end{array}$^–M*d*^$\begin{array}{c} n \end{array}$^, in order to compensate for the <85% of the M*a*^$\begin{array}{c} 2 \end{array}$^–M*a*^$\begin{array}{c} 4 \end{array}$^ relationship. Indeed, M*b*^$\begin{array}{c} 2 \end{array}$^ and M*d*^$\begin{array}{c} 1 \end{array}$^ are replicated (as their symbology implies) without mutation (= 100% relation).

Table 2 shows PID values for each museme allele-class in “The Two Brothers”, comparing alleles of M*a*–M*d* against others in the same allele-class^21^.

**Table 2:** PID values for museme alleles in “The Two Brothers”.

	**M*a*^$\begin{array}{c} 1 \end{array}$^**	**M*a*^$\begin{array}{c} 2 \end{array}$^**	**M*a*^$\begin{array}{c} 3 \end{array}$^**	**M*a*^$\begin{array}{c} 4 \end{array}$^**	**M*b*^$\begin{array}{c} 1 \end{array}$^**	**M*b*^$\begin{array}{c} 2 \end{array}$^**	**M*c*^$\begin{array}{c} 1 \end{array}$^**	**M*c*^$\begin{array}{c} 2 \end{array}$^**	**M*c*^$\begin{array}{c} 3 \end{array}$^**	**M*c*^$\begin{array}{c} 4 \end{array}$^**	**M*d*^$\begin{array}{c} 1 \end{array}$^**	**M*d*^$\begin{array}{c} 2 \end{array}$^**	**M*d*^$\begin{array}{c} 3 \end{array}$^**	**M*d*^$\begin{array}{c} 4 \end{array}$^**
M*a*^$\begin{array}{c} 1 \end{array}$^		85.7	100	57.1		66.7								
M*a*^$\begin{array}{c} 2 \end{array}$^			75.0	71.4										
M*a*^$\begin{array}{c} 3 \end{array}$^				57.1										
M*a*^$\begin{array}{c} 4 \end{array}$^														
M*b*^$\begin{array}{c} 1 \end{array}$^						80								
M*b*^$\begin{array}{c} 2 \end{array}$^														
M*c*^$\begin{array}{c} 1 \end{array}$^								28.6	85.7	14.3				
M*c*^$\begin{array}{c} 2 \end{array}$^									42.9	57.1				
M*c*^$\begin{array}{c} 3 \end{array}$^										14.3				
M*c*^$\begin{array}{c} 4 \end{array}$^														
M*d*^$\begin{array}{c} 1 \end{array}$^												50	83.3	83.3
M*d*^$\begin{array}{c} 2 \end{array}$^													83.3	50
M*d*^$\begin{array}{c} 3 \end{array}$^														83.3
M*d*^$\begin{array}{c} 4 \end{array}$^														

Without the anchor of the sequential-mapping constraint, many of these patterns would not, on the basis of their PID values, appear to be related. The similarities between M*a*^$\begin{array}{c} 2 \end{array}$^ and M*a*^$\begin{array}{c} 4 \end{array}$^, for example, inhere in relatively tenuous pitch connections—the 28.6% “PnID” (Percent *non*-IDentity = 100%−71.4) puts quite an expanse of clear blue water between them. In the case of the M*c*^$\begin{array}{c} 1 \end{array}$^–M*c*^$\begin{array}{c} 4 \end{array}$^ relationship, the considerably smaller 14.3% PID value (and therefore considerably greater 85.7% PnID) would not even suggest membership of the same allele-class^22^. In both cases, and as is often the case in musemic similarity relationships, it is the rhythm, contour and harmonic implication—the latter a prolongation of the tonic and dominant chords, respectively (Figure 1Bv)—which additionally binds these alleles together (and which would have to suffice in the absence of the sequential-mapping constraint). In the case of M*a*^$\begin{array}{c} 2 \end{array}$^ and M*a*^$\begin{array}{c} 4 \end{array}$^, the rise from the initial c^1^ to the apical a^1^ in b. 2 followed by a fall to the dominant g^1^ at the end of the first half-phrase is the common, unifying contour feature of the allele-class.

Measures of similarity have a bearing on the related issues of museme transmission and of museme resolution/subdivision. In general, cultural transmission is significantly more error-prone (in an informational sense) than biological transmission, so it may be presumed that most inter-museme PID values will be lower than 100%^23^. Below a certain context-specific threshold, a low PID value might be taken as evidence that any similarities are the consequences of homoplasy, not homology. But the converse may not always hold true: a very high PID might be associated with a pattern so generic and so commonplace that the two instances may have been independently generated (homoplasy), rather than directly transmitted (homology). In Cope's terms, such entities are “commonalities”: a category of “patterns which, by virtue of their simplicity—scales, triad outlines, and so on—appear everywhere. In a sense, commonalities seem to disappear in a sea of similarity” (Cope, 2003, p. 17). By contrast, and at the opposite end of a continuum of similarity categories (Jan, 2014, p. 4, Figure 1), longer and more distinctive patterns are termed “quotations”: a category which “often involve exact note and/or rhythm duplication” (Cope, 2003, p. 11). Quotations are more likely than commonalities to be homologous as opposed to homoplasious, and *vice versa*. Thus, one must also take into consideration the issue of museme length, in addition to the PID value, when attempting to determine whether two coindexes are related by homology or by homoplasy.

On this last point, and as noted in section 2, museme perception and cognition is contingent upon both bottom-up and top-down processing. The former to some extent tracks the sonic-acoustic regularities governed by the laws of physics. Given that these regularities include the harmonic series, it is perhaps not surprising that certain musical structures derived from this series—triads and particular (5–7-note, unequal-interval) scale-types—are common across many (but not all) musical cultures (Patel, 2008, pp. 19–21). Such structures are thus to some extent acoustically privileged and will (*ceteris paribus*) naturally constitute the “connective tissue”, the commonalities, of much music—which is not to say that the particular (rhythmic/harmonic) form they take in a given piece of music is not derived (memetically) from a specific antecedent coindex. Moreover, such commonalities are often useful in expediting the connection of more “characteristic” musemes (i.e., those closer to the “quotations” end than the “commonalities” end of Cope's (2003) continuum) and, in this capacity, they therefore serve as evolutionary “good tricks” (Dennett, 1995, pp. 77–78).

As a further complication, similarity values are often not helpful in trying to order musemes chronologically/sequentially in a nexus of transmission. As will be discussed further in section 4, evolution is not invariably associated with increasing complexity, however measured; in certain circumstances, adaptation might result in decreasing complexity. Moreover, the PID value measures editorial *differences* (it is not, strictly, an edit-distance metric; Levenshtein, 1966), which might result in no net change in absolute or relative complexity between two or more musemes; nor does it indicate the *direction* of change (toward greater simplicity or greater complexity), so a high PID might be associated with operations which result in the simplification of a museme, such as occurs between M*a*^$\begin{array}{c} 2 \end{array}$^ and M*a*^$\begin{array}{c} 4 \end{array}$^. Of course, this relationship is only one of simplification if M*a*^$\begin{array}{c} 2 \end{array}$^ is regarded as the antecedent and M*a*^$\begin{array}{c} 4 \end{array}$^ as the consequent; seen the other way round, it is a process of increasing complexity. If evolution were *only* taken to be a process of increasing complexity, then M*a*^$\begin{array}{c} 4 \end{array}$^ would be a candidate for the antecedent of M*a*^$\begin{array}{c} 2 \end{array}$^—which it might nevertheless still be, even though this specific (simplicity-complexity) justification is invalid.

Hitherto, these alleles have been treated as unitary, but if we hypothesize that three notes is the realistic lower threshold for a melodic museme to have perceptual-cognitive validity (Jan, 2007, p. 61), then the a^1^–a^1^–g^1^ melodic triad of b. 2 is the only common contiguous element between M*a*^$\begin{array}{c} 2 \end{array}$^ and M*a*^$\begin{array}{c} 4 \end{array}$^. (One might, however, regard Musemes M*a*^$\begin{array}{c} 2 \end{array}$^ and M*a*^$\begin{array}{c} 4 \end{array}$^ as being identical at the shallow-middleground level—having a c^1^–a^1^–g^1^ structure; but a full consideration of the structural-hierarchic location of the musemes under consideration is beyond the scope of the present article.) The first part of the museme—(c^1^)–c^1^–c^1^–e^1^–g^1^ in M*a*^$\begin{array}{c} 2 \end{array}$^, []–c^1^–c^1^–c^1^–c^1^ in M*a*^$\begin{array}{c} 4 \end{array}$^—is sufficiently dissimilar (despite the two common c^1^s) for one to envisage various scenarios to account for the etiology of the material of bb. 1–2 in these two song-variants, scenarios which may be generalized to other musemes in these six melodies and, indeed, more widely.

To contextualize these scenarios, it is useful to make a distinction between two ways of viewing these melodies and the alleles which constitute them, which might be conceived as extreme points on a “continuum of influence”. On the one hand (the imaginary left-hand (“closed”) side of the continuum), one could see these six melodies as an essentially secure ecosystem, impervious to perturbation by musemes external to its constituent allele classes. On the other hand (the imaginary right-hand (“open”) side of the continuum), one could see them as entirely receptive to influence by external factors (immigration of, or influence by, external musemes). In the case of “The Two Brothers”, it seems sensible to ascribe priority to intra-tune-family relationships, given the nature of this repertoire's transmission, while not ruling out the possibility that musemes from other sources—other tune-families, other repertoires—might have influenced the transmission relationships within this group of six melodic variants. It is also important to note that in such repertoires as the folk ballad there is obviously textual as well as musical replication, but this does not necessarily guarantee that, when a textual phrase is replicated from one context to another, the museme associated with the earlier text is the source of that associated with the later text—as other instances of “The Two Brothers” tune-family attest.

**Figure 2:**
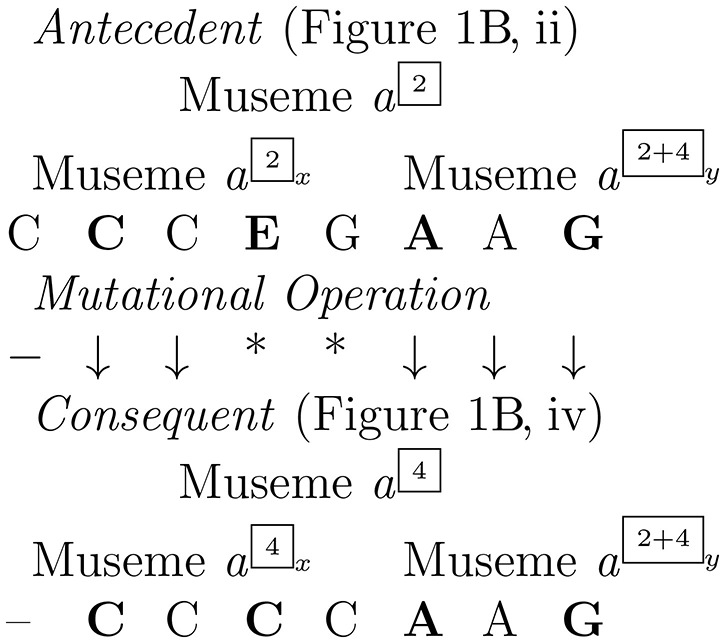
Museme *a*^$\begin{array}{c} 2 \end{array}$^–*a*^$\begin{array}{c} 4 \end{array}$^.

For M*a*^$\begin{array}{c} n \end{array}$^ and the multitude of comparable cases:

1. One could regard bb. 1–2 of “The Two Brothers” as consisting of only *one* museme (M*a*^$\begin{array}{c} 2 \end{array}$^ and M*a*^$\begin{array}{c} 4 \end{array}$^). If so, then given the similarities between the second halves of each variant (the a^1^–a^1^–g^1^ triad), which act as a kind of “anchor” (and given, of course, the sequential-mapping constraint), one would take the first halves, b. 1, as being edit-heavy, homology-associated mutations: to get from the antecedent to the consequent form (whichever is which), a fair amount of “earth moving” is required (Typke et al., 2007; see also Jan, 2014).
2. Alternatively, as shown in Figure 2, one could regard bb. 1–2 as consisting of *two* musemes (or two museme allele-classes), which one might label M*a*^$\begin{array}{c} 2 \end{array}$*x*^/M*a*^$\begin{array}{c} 4 \end{array}$*x*^ and M*a*^$\begin{array}{c} 2+4 \end{array}$*y*^ (the latter being the a^1^–a^1^–g^1^ triad). Under this alternative interpretation, then:
  - Liberated from their evolutionary dependency with M*a*^$\begin{array}{c} 2+4 \end{array}$*y*^, the relationship between M*a*^$\begin{array}{c} 2 \end{array}$*x*^ and M*a*^$\begin{array}{c} 4 \end{array}$*x*^ could be one of either homology (i.e., M*a*^$\begin{array}{c} 4 \end{array}$*x*^ is copied from M*a*^$\begin{array}{c} 2 \end{array}$*x*^ (or *vice versa*)) or homoplasy (i.e., M*a*^$\begin{array}{c} 4 \end{array}$*x*^ is copied from an antecedent other than M*a*^$\begin{array}{c} 2 \end{array}$*x*^). Homology might be more likely to be the case if one were situated on the left-hand/closed side of the “continuum of influence” referred to above; and homoplasy might be more likely to be the case if one were situated on the right-hand/open side of the continuum.
  - Given its relative brevity, the same qualification as to homology *versus* homoplasy applies to M*a*^$\begin{array}{c} 2+4 \end{array}$*y*^, which is a commonality (in Cope's (2003) terms) of tonal music. Thus, while perhaps unlikely on account of the wider melodic similarities, it could in principle be the case that both M*a*^$\begin{array}{c} 2\text{ or }4 \end{array}$*x*^ and M*a*^$\begin{array}{c} 2+4 \end{array}$*y*^ are separately transmitted to the consequent of “The Two Brothers”, circumventing the posited antecedent.
3. For all these scenarios, some degree of blending inheritance might have occurred: positioned in the center of the continuum of influence, an intra-tune-family transmission event might have been influenced by an extra-tune-family factor. Thus, if Figure 1Bii were antecedent to Figure 1Biv then replication of the latter might have been meditated by the memory of a melody containing a repeated-note museme.

Given that Table 2 shows intra-museme-allele-class PID values, what is not considered are inter-museme-allele-class values. One of the latter is, however, shown (italicized), namely that between M*a*^$\begin{array}{c} 1 \end{array}$^ and M*b*^$\begin{array}{c} 2 \end{array}$^, the relatively high value of 66.7% (higher, of course, than some intra-museme-allele-class values) indicating the presence of rhyme/symmetry within the first half of the melody^24^. The higher the intra-museme-allele-class (“vertical”) PID values of any tune-family, the greater the perceived *synchronic* unity (its coherence as a collection of melodies) of the family; whereas the higher the inter-museme-allele-class (“horizontal”) values of any individual melody, the greater the perceived *diachronic* unity (its coherence as a collection of musemes) of that melody—and *vice versa*. Both forms of unity might act as musemic selection pressures: the higher the perceived unity, synchronic or diachronic, the easier it is for listeners and singers to remember these melodies and therefore the more evolutionarily successful their constituent musemes may tend to be, if success is measured in terms of the number of copies of a given museme in a museme-pool. This selection pressure might be operative in many musemeplexes, and might be a factor driving the musemic collaboration which gives rise to them.

## 4. Phylomemetics and cultural taxonomies

The reference to “phylogenetic analysis” in the quotation in section 2 (page 5) is significant, in that just as the long-term outcomes of biological selection can be represented in terms of branching lineages on (by convention) a tree diagram—where species bifurcate to give rise to sub-species, etc. (Darwin, 2008, p. 90)—so can those of cultural evolution. In the case of the group of museme alleles constituting the particular subset of “The Two Brothers” tune-family shown in Figure 1, one might apply the principles of cladistic taxonomy (Hennig, 1999) to arrive at a representation, a *cladogram*, not of the evolutionary relationships between “dialects” (arguably the cultural equivalent of species; Meyer, 1996, p. 23), but between musemes (the cultural equivalent of genes)^25^. Thus, this enterprise is closer to molecular genetics than it is to species taxonomy.

As a first word of caution, attempting to calculate cultural phylogenies—what might be termed *phylomemies*—from such a small group of short melodies risks falling foul of what might be termed the distinction between *real* and *virtual* phylogen/memies. A real phylogen/memy is one which is objectively evolutionarily correct, indicating the transmission relationships between the replicators at various positions on the cladogram. A virtual phylogen/memy is one which arrives (perhaps as a consequence of a restricted sample size) at a “pseudo-cladogram” which, while a logical and (perhaps more importantly) parsimonious representation of the patterns under investigation, is nevertheless (potentially) *not* evolutionarily true (and is therefore not properly cladistic) because it does not take into account patterning “external” to the sample under consideration. This external patterning, if included, might alter the relationships represented by the cladogram. It would appear considerably easier to arrive at a real phylogeny (where groups of potentially related organisms are often relatively geographically localized, morphologically distinct and, nowadays, genetically tractable) than it is to arrive at a real phylomemy (where groups of potentially related cultural forms are often scattered across space and time).

Yet this enterprise is worth pursuing, if only to illustrate the possibilities of the approach, one which Howe and Windram (2011) term “phylomemetics”, the cultural equivalent of phylogenetics. As they acknowledge (Howe and Windram, 2011, p. 1), this is by no means a new methodology in the humanities, where philologists in both linguistic and musical research have long attempted to reconstruct *stemmata* showing relationships of transmission and mutation in sources as diverse as biblical texts and medieval music manuscripts (Cook, 2015). Conducted under (or, some might fear, annexed by) the rubric of phylomemetics, such research can incorporate all the intellectual infrastructure of Darwinism—the notions of variation, replication and selection; concepts of fitness; and ideas of lineage bifurcation and divergence—in tracing connections between the phenomena under investigation^26^.

**Figure 3:**
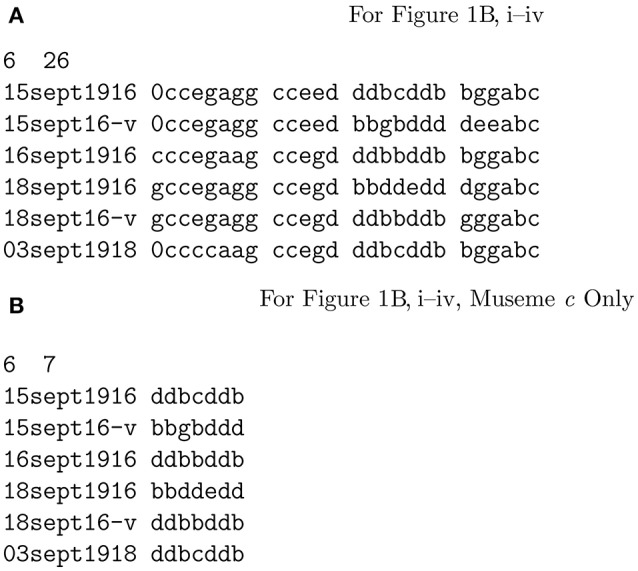
Input data for “The Two Brothers”.

**Figure 4:**
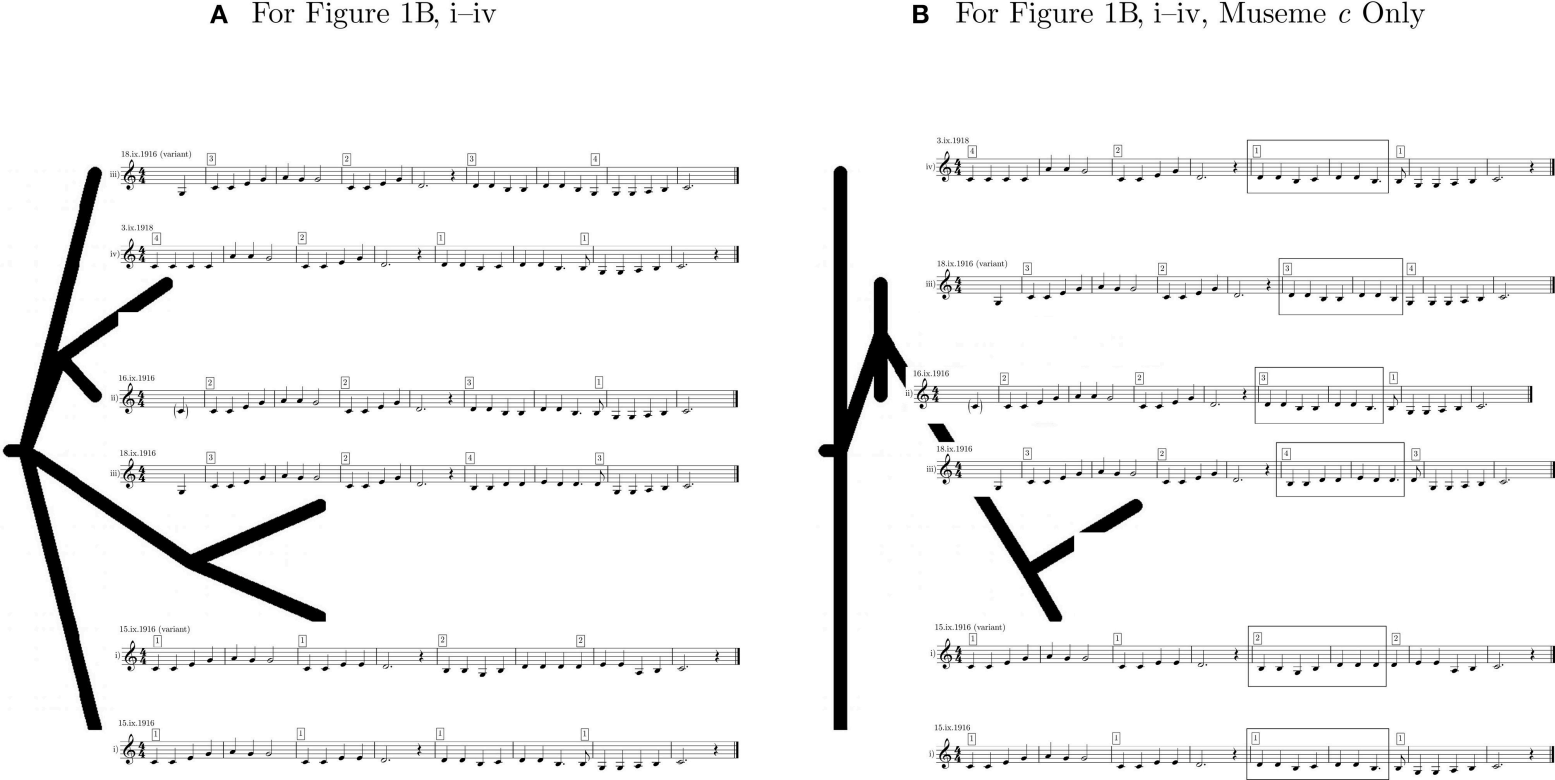
Output phylomemetic trees of “The Two Brothers”.

Using the phylogeny-calculation software *Phylip* (Felsenstein, 2016), the six forms of “The Two Brothers” in Figure 1B were analyzed. This used the input file shown in Figure 3A, which is a date-ordered list—based on Figure 1B and in which “v” represents the variant forms of Figures 1Bi,iii—of the melodies consisting of a sequence of their constituent pitches, grouped into museme alleles^27^. It should be stressed that this is an illustrative calculation only, designed to outline a methodology which might be adopted (as discussed in section 5) in larger studies. The highly restricted dataset naturally limits the scope of the conclusions that can be drawn. The phylomemetic tree shown in Figure 4A was generated using the *Pars* utility, which “is a general parsimony program which carries out the Wagner parsimony method (Eck and Dayhoff, 1966) with multiple states. Wagner parsimony allows changes among all states. The criterion is to find the tree which requires the minimum number of changes” (Felsenstein, 2016). For ease of comparison, the text-based output of *Pars* (strictly, that of the graphics-generating utility *DrawGram*) has been replaced in Figure 4 by images of the relevant melodies^28^.

Such cladograms represent descent with modification, whereby items located to the left (bottom/past) are hypothesized to be evolutionarily earlier than those located to the right (top/present), and where proximity to points of bifurcation (branch-length) represents relative evolutionary distance. While parsimony does not invariably align with evolutionary reality (a parsimonious tree is not necessarily a “real” tree, in terms of the binarism referred to above), it is a powerful constraint on evolutionary possibilities. Given this, it is reasonable to infer that both real and virtual lineages will generally proceed from left to right by the minimal mutational distances (this is not to deny the possibility of more radical, saltational, change). As suggested in section 3, evolution is fundamentally a process of *adaptive change* (Ridley, 2004, p. 4) and not necessarily one where that change leads to an increase in “the logarithm of the total information content of the biosystem (genes plus memes)” (Ball, 1984, p. 154)^29^. In the light of this, and of the proviso made in section 3 that date of collection does not necessarily align with the evolutionary chronology of these melodies, one must reiterate that, when undertaking phylomemetic analysis, melodic simplicity does not necessarily correlate with chronological anteriority, any more than melodic complexity corresponds with chronological posteriority.

As a second word of caution—one which applies more broadly to any attempt to analyse music by means of the kinds of symbolic representations used in *Phylip*—in order to perform the phylomemetic analysis, the musical patterning of these songs, already converted to their traditional western letter-name notation in Figure 1, was rendered as a series of ASCII characters to form the input to *Pars*. In this way, the melodies of these ballads are treated as a text. This means that the analysis is operating on a representation two stages removed from the living performances recorded over a century ago: not only has the rendition been regularized and shoehorned into western notation, a form of “lossy” compression; but this representation has itself been further divorced from its connection with sound by its reduction to a mere symbol-set, an abstract series of M*x*^$\begin{array}{c} n \end{array}$^ patterns. Perhaps more fundamentally, while the *Phylip* software to some extent “understands” genetics, in that it is based on a formalization of the dynamics of the biochemistry underpinning it, it has little conception of music and the dynamics of pitch and rhythm combination underpinning it. Nevertheless, the symbols offered as input bear at least some connection with their long-distant musical antecedents, and so permit a provisional phylomemetic analysis based on parsimony relationships to be conducted.

In addition to analyzing relationships between song melodies as a whole, this type of analysis may also be conducted at the level of the museme allele, as represented in Figures 3B, 4B, which show only the four alleles of M*c*. Importantly, if cladograms generated from complete song melodies are different from those derived from specific museme alleles within a melody, then this affords evidence in support of the second claim, made in section 3: that statistical data derived from measuring mutational changes, while illuminating, are epiphenomena of musemic evolution.

While there are many complex relationships represented within the cladograms of Figure 4, not all of which can be elaborated upon here, the following points may be made in summary (again reiterating that the *Pars* utility is operating on a deprecated, symbolic representation of music without any knowledge of music theory):

1. In Figure 4A, the melodies shown in Figures 1Bi,iii (variant) are hypothesized to be evolutionarily prior and are distinguished by the difference between M*b*^$\begin{array}{c} 1 \end{array}$^ and M*b*^$\begin{array}{c} 2 \end{array}$^ and by a pitch difference between M*c*^$\begin{array}{c} 1 \end{array}$^ and M*c*^$\begin{array}{c} 3 \end{array}$^.
2. In the same cladogram, two groupings of posited evolutionary descendants link Figures 1Bii,iv (perhaps by virtue of the common a^1^–a^1^–g^1^ melodic triad in M*a*^$\begin{array}{c} 2 \end{array}$^ and M*a*^$\begin{array}{c} 4 \end{array}$^ (designated earlier as M*a*^$\begin{array}{c} 2+4 \end{array}$*y*^)); and Figure 1Bi (variant) and Figure 1Biii (perhaps by virtue of the common a^1^–g^1^–g^1^ melodic triad in M*a*^$\begin{array}{c} 1 \end{array}$^ and M*a*^$\begin{array}{c} 3 \end{array}$^ (which might, by extension with M*a*^$\begin{array}{c} 2+4 \end{array}$*y*^, be designated M*a*^$\begin{array}{c} 1+3 \end{array}$*y*^), and (in the same pair) of the prominence of the pitch d^1^ toward the end of M*c*^$\begin{array}{c} 2 \end{array}$^ and M*c*^$\begin{array}{c} 4 \end{array}$^).
3. In terms of chronology, this first cladogram broadly aligns with the dates of collection of these songs; but, as noted in the provisos above—date of collection ≠ date of origin; simplicity/complexity ≠ anteriority/posteriority, respectively—this cladogram can only offer circumstantial evidence. Indeed, the evolutionarily later placement of Figure 1Biv (with its arguably most basic form of M*a*, M*a*^$\begin{array}{c} 4 \end{array}$^) broadly accords with the assertion that simplicity/complexity ≠ anteriority/posteriority.
4. In Figure 4B, the exclusive focus on M*c* motivates a restructuring of the cladogram, in that parsimonious relationships of similarity between the alleles of this museme do not always align with parsimonious relationships of similarity between the melodies as a whole (Figure 4A). As an example, M*c*^$\begin{array}{c} 1 \end{array}$^ is represented as evolutionarily prior to the three other alleles of M*c*, giving Figures 1Bi,iv priority; but, in Figure 4A, the evolutionarily prior melodies are Figures 1Bi,iii (variant). This indeed affords evidence in support of the second claim: that statistical data derived from measuring mutational changes (Figure 4A) are epiphenomena of musemic evolution (Figure 4B), because M*c* (and indeed any museme) is arguably more meaningful—perceptually-cognitively and evolutionarily—than the larger melody of which it forms a part.
5. In terms of chronology, this second cladogram is (quasi-) anachronistic, in that it ascribes evolutionary (co-)primacy to the “latest” (and also “earliest”) of these musemes, M*c*^$\begin{array}{c} 1 \end{array}$^. As specified by the provisos in the third (“chronology”) point above, this cladogram does not constitute hard evidence in favor of a phylomemy which runs counter to the collection-date ordering.

This consideration has only scratched the surface of the complex relationships inherent in Figure 4, itself only a small case study. For one thing, while these melodies would normally have been performed unaccompanied, their implied harmony (Figure 1Bv) may have acted as a selection pressure^30^. Given the tendency for harmonic changes to coincide with points of metrical accentuation—Temperley's “HPR [Harmonic Preference Rule] 2 (Strong Beat Rule)” (Temperley, 2001, p. 151)—it may be the case that M*a*^$\begin{array}{c} 1+3 \end{array}$*y*^, with their implied shift to the tonic chord on the second (weak) rather than the third (strong) crotchet beat of the bar (as in M*a*^$\begin{array}{c} 2+4 \end{array}$*y*^), have either a selective advantage or (paradoxically) a selective *dis*advantage, depending on context^31^.

But the overriding issue here is that the dichotomy identified above between real and virtual phylomemies is clearly problematic, for while Savage and Atkinson (2015, p. 167) are laudable in their injunction that statistical-phylomemetic analysis is (only) a stepping stone toward the understanding of “higher-level units of musical structure and meaning”, the statistical data—even considered in conjunction with musemic organization—does not always permit the reconstruction of higher-level-unit phylomemies with any real certainty, as is demonstrated by the present study. Perhaps we might simply hypothesize that, in the absence of detailed knowledge of the transmission events under investigation, the cladograms in Figure 4 *predict* the true temporal ordering of (phrase- or museme-level) events. Thus, we are taking the most parsimonious phylomemy to be the most plausible, and assuming that, when the historical record is obscure, this criterion should be primary when attempting to reconstruct cultural-evolutionary histories.

## 5. Conclusion: two brothers?

While the lyrics of “The Two Brothers” are decidedly grim, the spirit of this article is optimistic, in that it holds that perceptual-cognitive and statistical models of musical evolution are also brothers (or sisters), and that—unlike the ballad texts—they can go on not to do violence to each other but to grow together and to complement each other, developing to be cooperative adults working for a two-fold common cause: the understanding of cultural evolution as a subset of a wider Darwinian view; and the development of methodologies along the perceptual-cognitive–statistical continuum to investigate its operation.

To return to the two claims underpinning the argument here—(i) that a purely statistical approach based on counting note-edits without consideration of perceptual-cognitive aspects gives an incomplete account of cultural evolution; and (ii) that statistical data derived from measuring mutational changes, while illuminating, are epiphenomena of musemic evolution—we might assert that both have been supported by the (admittedly limited) case study outlined here. That is [apropos claim (i)], Savage's (2017) statistical data on “The Two Brothers” are arguably contextualized, enriched and elucidated by considering the musemic structure of the tune-family music-analytically, music-psychologically and music-phylomemetically; and [apropos claim (ii)] the discussion conducted under the third of these rubrics suggests a strong regulatory role for museme-level (as opposed to note-level) processes.

This case study—a small-scale empirical example of how to pursue a novel methodological strategy—is arguably scalable (by means of more systematic use of computer technology) in ways which would foster perceptual-cognitive–statistical collaboration in research on cultural evolution. The methodology for this, which is essentially a formalization and expansion of what is discussed here, is summarized as follows. As will be clear, many of the relevant technologies already exist and so, as is often the case with advances in research, it is largely a matter of synergistic interconnection for this to become a reality.

1. Music databases need to be utilized. To maximize the big-data approach, sizeable databases in an established music-encoding format should be employed (Selfridge-Field, 1997). The *Humdrum Toolkit*'s (Huron, 2002) ^**^kern format is used for several databases, including the Essen Folksong Collection (Schaffrath, 1995), together with various art-music repertoires, and this format can be translated to other encodings, such as MusicXML (MakeMusic, 2016).
2. Algorithms need to be developed to segment and interrogate the encodings in 1 above in order to locate patterns which are (i) perceptually-cognitively meaningful (using criteria drawn from the music-cognition and music-theory literature); and (ii) replicated in two or more contexts—i.e., patterns which satisfy the necessary conditions for existing as musemes. In addition to Savage's (2017) software, many such algorithms for segmentation and pattern-matching have been developed over recent years, often under the stimulus of the aforementioned MIREX project (Lartillot, 2009; Conklin, 2010; Velardo et al., 2016).
3. The outputs of 2 above need to be processed with phylogenetic software in order to reconstruct hypothetical phylomemies of musemes and the works of which they form part. To accomplish this, greater formalization is needed for the encoding of musical elements and for their incorporation into software designed primarily for (biological) phylogenies. For one thing, a ^**^kern/MusicXML–*Phylip* converter might usefully be developed.
4. Prosopographic analysis (Keats-Rohan, 2007), which is a nascent research methodology in historical musicology, could be extended as a means of contextualizing and assigning probabilities to the outputs of 3 above.

While the four points above seem clear in outline, their connection is likely to prove difficult to implement in practice, given the recalcitrant complexity of music and the intricacy of the programming tasks required. Yet success in this venture offers a rich promise: that of reconstructing how music may have been perceived and transmitted across time and place in various human societies; and therefore of offering synchronic overviews and simulacra of once-vibrant, diachronic musical cultures.

## Author contributions

The author confirms being the sole contributor of this work and approved it for publication.

### Conflict of interest statement

The author declares that the research was conducted in the absence of any commercial or financial relationships that could be construed as a potential conflict of interest.

## Footnotes

^1^I am grateful to Valerio Velardo, Alexey Nikolsky, and the anonymous reviewers for their helpful comments on earlier versions of this article.

^2^In eastern Europe, there has arguably been a more thoroughgoing synthesis, the integration of artistic/humanistic and scientific methodologies being long established, for example, in Russian scholarship (Grigoryan, 2011).

^3^http://www.music-ir.org/mirex/wiki/MIREX_HOME

^4^See also the *Journal of Creative Music Systems* (http://jcms.org.uk/).

^5^It should be remembered that the (musicological) conception of music as a series of discrete structures/objects (works, Goehr, 1992) produced by named and celebrated author-composers and notated unambiguously is a relatively recent western-European phenomenon, and that most human music is (from an *ethno*musicological standpoint) communal, processive and deeply enmeshed with other media, such as dance and poetry, and with worship. In Taruskin's (1995) phrase, this is the distinction between music as text and music as act.

^6^Because the evolutionary algorithm (Dennett, 1995, p. 343) is substrate-neutral, it makes little sense to distinguish between “natural” and “cultural” selection—this being the principle underpinning Universal Darwinism (Dawkins, 1983b).

^7^Thus, and at the risk of multiplying terminology, I use “phemotype” as the memetic counterpart to the genetic “phenotype”. By extension, I use “memotype” as the counterpart to “genotype” (Jan, 2007, p. 30, Table 2.1).

^8^Thus, it takes as its starting point the assumptions that (i) culture evolves; (ii) that this evolution is broadly Darwinian; and (iii) that memetics offers the best formalization of this cultural-evolutionary process.

^9^One might draw a distinction between formalization and quantitative studies: the former is an abstract attempt to theorize the terrain and dynamics of a system; the latter is a concrete attempt to measure a system using various metrics, perhaps using some formalization as a guide.

^10^These are not absolute correlations, but simply attempts to align phenomena at analogous structural levels within their parent “ontological category” (Velardo, 2016, p. 104, Figure 3).

^11^I alighted upon the term “museme” independently of Tagg (2016), conflating “music” and “meme” in an example of convergent evolution (*homoplasy*; see also section 3). While there are alignments between our uses of the term, mine is distinguished from Tagg's by its specifically evolutionary, as opposed to semiotic, focus—as a unit of cultural selection in music.

^12^Verses 4 and 6 of one variant of this ballad read: “4: Brother took out his little penknife, / It was sharp and keen. / He stuck it in his own brother's heart, / It caused a deadly wound. 6: He buried his bible at his head, / His hymn book at his feet, / His bow and arrow by his side, / And now he's fast asleep.” (Bronson, 1959, p. 391).

^13^The segmentation of these melodies is largely unproblematic, being guided by Gestalt-psychological segmentation criteria (Deutsch, 1999; Snyder, 2000). While a phrase is not necessarily the same as a museme, in the case of this melody the four short phrases are. The distinctive .–♪ rhythm straddling M*c*–M*d* in most of these melodies also acts (residually) in the two examples where the junction is –, i.e., the variant forms of Figures 1Bi,iii.

^14^While Bronson categorizes these six as belonging to “Group B” of this tune-collection (Bronson, 1959, pp. 387–393, nos. 9–20), others in this group are often significantly different to the homogeneous six which are shown in Figure 1B.

^15^Such similarities suggests a deep commonality between song and dance melodies arising from the imperatives of symmetry, balance and an arch-shaped (low-high-low) tension-curve.

^16^Such numeration is, naturally, undertaken “vertically” (i.e., as an *intra*-museme-allele-class system), and not “horizontally” (i.e., as an *inter*-museme system). The latter approach would indicate a degree of similarity, for example, between M*a*^$\begin{array}{c} 3 \end{array}$^ and M*c*^$\begin{array}{c} 4 \end{array}$^ (Figure 1Biii). Nevertheless, this particular connection is a *contour*-based similarity, and not one inhering in the *intervallic* and *scale-degree* recurrences which I employ here to define similarities and differences between musemes and their alleles.

^17^While Savage (2017, p. 51) argues for, and operationalises, the primacy of pitch over rhythm in his melodic-similarity determinations—yet usefully takes into account the distinction between accented and unaccented pitches—future research in this area might usefully integrate both parameters in a more sophisticated PID metric.

^18^This attribute of independent replication is assumed for the sake of argument, but it is not difficult to envisage easily finding *coindexes* (Jan, 2007, p. 71) of the individual musemes of “The Two Brothers”, replicated separately from the assemblage of which they form a part in the ballad.

^19^A PID <85% may still indicate a relationship of (partial) transmission, in which one or more musemes from one melody are assimilated by another, largely dissimilar, melody.

^20^This is a consequence of the phenomenon famously summed up by the comedian Eric Morecambe, who said to André Previn—after a shambolic start by Morecambe to Grieg's Piano Concerto in A minor—“I'm playing all the right notes—but not necessarily in the right order” (McCann, 1999, p. 234).

^21^The bracketed anacrusis c^1^ (Bronson, 1959, p. 391, no. 16) in Figure 1Bii is included here, as it is in Savage's mutation calculation, represented in Figure 1A.

^22^Perhaps criteria might be devised which would conclude that they are not actually in the same allele-class, or that they are only members of an “allele-super-class”, perhaps one defined by harmony but not including scale-degree factors. While the present focus is largely upon melodic (linear pitch plus rhythm) patterning, one could vary the number of parameters taken into consideration in order to narrow or broaden the definition of a museme. In this way, a museme would be seen as a multiparametric complex (a “style structure”, in Narmour's terminology) made up of several uniparametric simplexes (“style forms/shapes”) (Narmour, 1977, pp. 173–174; 1990, p. 34), although this runs the risk of blurring the distinction, if one truly exists, between a museme and a musemeplex.

^23^One concomitant of the dichotomy expressed in Note 7 is that a work-centric view of music attempted, until relatively recently, to enforce a single correct and objective text, whereas a process-centric view accepts the diversity of different acts, be these interpretations of “classical” works or variants of folk musics.

^24^Bronson argues for the primacy of musical over textual rhyme (Bronson, 1959, p. xii).

^25^There are various different approaches to taxonomy, and biologists often argue testily as to their relative merits—in Dawkins' view, taxonomy is “one of the most rancorously ill-tempered of biological fields. Stephen [Jay] Gould has well characterized it with the phrase ‘names and nastiness’” (Dawkins, 2006, p. 275). But a cladistic approach, particularly one where genetic evidence is employed, is the one most likely to be evolutionarily “correct” in biological taxonomy (Ridley, 2004, p. 489).

^26^It might be argued that phylomemies differ from phylogenies in their potential for “cross-fertilization”, whereby two lineages may share material, or even rejoin, after bifurcation. But this is also true, perhaps to a lesser extent, in nature, where gene-transfer between recently bifurcated lineages remains possible for a limited time.

^27^This might be further developed by incorporating rhythmic values, whereby “bbb” = . and “b” = ♪.

^28^Note that these are “rooted” phylomemies: there is assumed to be an unidentified common ancestor to the left of the tree (Ridley, 2004, p. 439).

^29^This may often be the case with oral transmission, where the principle of *lectio difficilior potior*—“the more difficult reading is the stronger” (Robinson, 2001)—might support one in ascribing chronological anteriority to a more complex form.

^30^Given that unaccompanied melodies in western music normally have clear harmonic implications (a phenomenon arguably most richly developed in the solo violin music of J.S. Bach), the perceptual-cognitive salience of mutations will tend to be evaluated in the light of the silent musemes constituting the underpinning chord progressions. Implied harmony therefore constitutes a selection pressure because it motivates an assessment of the altered conformity of (elements of) a mutant museme with the associated chord vis-à-vis the alignment of its antecedent. In non-western cultures, no such implicative coadaptation exists between melodic and harmonic musemes.

^31^This context includes the likelihood that, for some listeners, such harmonic-rhythm disruptions might be appealing (and therefore selectively advantageous from the museme's perspective), whereas for other listeners the opposite might be the case.
